# Limit of Riemann Solutions to the Nonsymmetric System of Keyfitz-Kranzer Type

**DOI:** 10.1155/2014/287256

**Published:** 2014-04-03

**Authors:** Lihui Guo, Gan Yin

**Affiliations:** College of Mathematics and System Sciences, Xinjiang University, Urumqi 830046, China

## Abstract

The limit of Riemann solutions to the nonsymmetric system of Keyfitz-Kranzer type with a scaled pressure is considered for both polytropic gas and generalized Chaplygin gas. In the former case, the delta shock wave can be obtained as the limit of shock wave and contact discontinuity when *u*_−_ > *u*_+_ and the parameter *ϵ* tends to zero. The point is, the delta shock wave is not the one of transport equations, which is obviously different from cases of some other systems such as Euler equations or relativistic Euler equations. For the generalized Chaplygin gas, unlike the polytropic or isothermal gas, there exists a certain critical value *ϵ*_2_ depending only on the Riemann initial data, such that when *ϵ* drops to *ϵ*_2_, the delta shock wave appears as *u*_−_ > *u*_+_, which is actually a delta solution of the same system in one critical case. Then as *ϵ* becomes smaller and goes to zero at last, the delta shock wave solution is the exact one of transport equations. Furthermore, the vacuum states and contact discontinuities can be obtained as the limit of Riemann solutions when *u*_−_ < *u*_+_ and *u*_−_ = *u*_+_, respectively.

## 1. Introduction

The nonsymmetric system of Keyfitz-Kranzer type can be written as

(1) $$\begin{array}{c} \rho_{t}+{\left(\rho \phi \left(\rho ,u_{1},u_{2},\ldots ,u_{n}\right)\right)}_{x}=0,\\ {\left(\rho u_{i}\right)}_{t}+{\left(\rho u_{i}\phi \left(\rho ,u_{1},u_{2},\ldots ,u_{n}\right)\right)}_{x},\;i=1,2,\ldots ,n, \end{array}$$

where

(2) $$\begin{array}{c} \phi \left(\rho ,u\right)=\phi \left(u\right)-p\left(\rho \right) \end{array}$$

is a nonlinear function. A more general form of system (1) was first derived as a model for the elastic string by Keyfitz and Kranzer [1].

When *n* = 1, *ϕ*(*ρ*, *u*) = *u* − *p*, and *p* = *p*(*ρ*), system (1) can be read as

(3) $$\begin{array}{c} \rho_{t}+{\left(\rho \left(u-p\right)\right)}_{x}=0,\\ {\left(\rho u\right)}_{t}+{\left(\rho u\left(u-p\right)\right)}_{x}=0. \end{array}$$

Let *u* = *v* + *p*; system (3) can be rewritten as the Aw-Rascle model [2]:

(4) $$\begin{array}{c} \rho_{t}+{\left(\rho v\right)}_{x}=0,\\ {\left(\rho \left(v+p\right)\right)}_{t}+{\left(\rho v\left(v+p\right)\right)}_{x}=0, \end{array}$$

where *ρ*, *v* represent the density and the velocity of cars on the roadway, respectively; the state equation *p*(*ρ*) = *ρ* ^*γ*^, *γ* > 0 is smooth and strictly increasing with

(5) $$\begin{array}{c} 2p^{\prime }\left(\rho \right)+\rho p^{\prime \prime }\left(\rho \right)>0\;\text{for}\;\;\rho >0. \end{array}$$

The Aw-Rascle model (4) resolves all the obvious inconsistencies and explains instabilities in car traffic flow, especially near the vacuum, that is, for light traffic with few slow drivers. In 2008, Berthelin et al. [3] studied the limit behavior which was investigated by changing *p* into *ϵp* and taking *p*(*ρ*) = (1/*ρ* − 1/ρ*), *ρ* ≤ ρ*, where ρ* is the maximal density which corresponds to a total traffic jam and is assumed to be a fixed constant although it should depend on the velocity in practice. Then, Shen and Sun [4] studied the limit behavior without the constraint of the maximal density, in which the delta shock and vacuum state were obtained through perturbing the pressure *p*(*ρ*) suitably.

For the nonsymmetric system of Keyfitz-Kranzer type (3), under the following two assumptions on *p*(*ρ*),

(6) $$\begin{array}{c} p\left(0\right)=0,\;\;\underset{\rho \to 0}{\lim}\rho p^{\prime }\left(\rho \right)=0,\\ \rho p^{\prime \prime }\left(\rho \right)+2p^{\prime }\left(\rho \right)>0\;\text{for}\;\;\rho >0,\\ \underset{\rho \to 0}{\lim}\rho p\left(\rho \right)=0,\;\;\underset{\rho \to \infty }{\lim}\rho p^{\prime }\left(\rho \right)\geq A,\\ \rho p^{\prime \prime }\left(\rho \right)+2p^{\prime }\left(\rho \right)>0\;\text{for}\;\;\rho >0, \end{array}$$

Lu [5] established the existence of global bounded weak solutions of the Cauchy problem by using the compensated compactness method. Recently, Lu [6] studied the existence of global entropy solutions to general system of Keyfitz-Kranzer type (3). In 2013, Cheng [7] considered the Riemann problem and two kinds of interactions of elementary waves for system (3) with the state equation for Chaplygin gas:

(7) $$\begin{array}{c} p\left(\rho \right)=-\frac{1}{\rho }. \end{array}$$

In this paper, our main purpose is to study the limit behavior of Riemann solutions to the nonsymmetric system of Keyfitz-Kranzer type (3) as the parameter *ϵ* goes to zero. In 2001, Li [8] was concerned with the limits of Riemann solutions to the compressed Euler equations for isothermal gas by letting the temperature go to zero. Then Chen and Liu [9, 10] presented the results of the compressible Euler equations as pressure vanishes. There are many results on the vanishing pressure limits of Riemann solutions; we refer readers to [4, 11–13] and the references cited therein for more details.

As the pressure vanishes, system (3) formally transforms into the so-called pressureless gas dynamics model or transport equations:

(8) $$\begin{array}{c} \rho_{t}+{\left(\rho u\right)}_{x}=0,\;\;{\left(\rho u\right)}_{t}+{\left(\rho u^{2}\right)}_{x}=0, \end{array}$$

where *ρ* and *u* stand for the density and the velocity of the gas, respectively. System (8) is also called zero-pressure gas dynamics. It can be derived from zero-pressure isentropic gas dynamics [14]. System (8) is referred to as the adhesion particle dynamics system to describe the motion process of free particles sticking under collision in the low temperature and the information of large-scale structure in the universe [15, 16]. It is easy to see that the delta shock and vacuum do occur in the Riemann solutions of (8); see [17]. We also refer readers to [4, 18–23] and the references cited therein for some results on delta shock waves.

By letting *p* be *ɛp*, system (3) can be changed to

(9) $$\begin{array}{c} \rho_{t}+{\left(\rho \left(u-\epsilon p\right)\right)}_{x}=0,\\ {\left(\rho u\right)}_{t}+{\left(\rho u\left(u-\epsilon p\right)\right)}_{x}=0. \end{array}$$

In the present paper, we focus on system (9) with equation of state for both polytropic gas and generalized Chaplygin gas. Firstly, we study limit of Riemann solutions to system (9) with the state equation

(10) $$\begin{array}{c} p\left(\rho \right)=\rho^{\gamma },\;\gamma >0, \end{array}$$

as *ϵ* tends to zero. If *u*_−_ > *u*_+_, we found that the Riemann solution tends to a delta shock wave solution when *ϵ* → 0. However, the propagating speed and the strength of the delta shock wave in the limit situation are different from the classical results of transport equations (8) with the same Riemann initial data. If *u*_−_ < *u*_+_, the Riemann solution tends to a two-contact discontinuity solution to the transport equations (8) as *ϵ* → 0. The intermediate state between the two-contact discontinuities is a vacuum state. When *u*_−_ = *u*_+_, the Riemann solutions converge to one-contact discontinuity solutions of system (8). Then, we investigate system (9) for generalized Chaplygin gas:

(11) $$\begin{array}{c} p\left(\rho \right)=-\rho^{-\alpha },\;0<\alpha \leq 1, \end{array}$$

where *α* = 1 is for Chaplygin gas. We find that, as *ϵ* arrives at a certain critical value *ϵ*_2_ depending only on the given Riemann initial data (*u*_±_, *ρ*_±_), the solution involving one shock and one contact discontinuity converges to a delta shock solution of system (9) and (11). Eventually, when *ϵ* tends to zero, the delta shock wave solution is exactly the solution of transport equations (8). Thus we can see that the process of delta shock wave formation is obviously different from those in [4, 8–13] and so forth.

The paper is organized as follows. In Section 2, we give some preliminary knowledge for system (8). In Section 3, we present the Riemann solutions to system (9). In Section 4, we display the limit of Riemann solutions to the nonsymmetric system of Keyfitz-Kranzer type (9).

## 2. The Riemann Solutions of System (8)

In this section, we briefly review the Riemann solutions of (8) with initial data:

(12) $$\begin{array}{c} \left(u\left(x,0\right),\rho \left(x,0\right)\right)=\left(u_{\pm },\rho_{\pm }\right),\;\pm x>0, \end{array}$$

where *ρ*_±_ > 0, the detailed study of which can be founded in [17].

Transport equations (8) have a double eigenvalue *λ* = *u* with only one corresponding right eigenvector *r* = (1,0)^*⊤*^. By simple calculation, we obtain ∇*λ* · *r* = 0, which means that system (8) is linearly degenerate.

Given any two constant states (*u*_±_, *ρ*_±_), we can constructively obtain the Riemann solutions of (8) and (12) containing contact discontinuities, vacuum, or delta shock wave.

For the case *u*_−_ < *u*_+_, the solution containing two contact discontinuities and a vacuum state can be expressed as

(13) $$\begin{array}{c} \left(u,\rho \right)\left(x,t\right)=\{\begin{array}{cc} \left(u_{-},\rho_{-}\right), & x\leq u_{-}t,\\ \left(\xi ,0\right), & u_{-}t\leq x\leq u_{+}t,\\ \left(u_{+},\rho_{+}\right), & x\geq u_{+}t. \end{array} \end{array}$$

For the case *u*_−_ = *u*_+_, we connect the constant states (*u*_±_, *ρ*_±_) by one contact discontinuity.

For the case *u*_−_ > *u*_+_, a solution containing a weighted *δ*-measure supported on a line will be constructed to connect the constant (*u*_±_, *ρ*_±_). So we define the solution in the sense of distributions as follows.

**Definition 1**

A pair (*u*, *ρ*) constitutes a solution of (8) in the sense of distributions if it satisfies

(14) $$\begin{array}{c} \overset{+\infty }{\underset{0}{\int }}\overset{+\infty }{\underset{-\infty }{\int }}\left(\rho \phi_{t}+\left(\rho u\right)\phi_{x}\right)\text{d}x\;\;\text{d}t=0,\\ \overset{+\infty }{\underset{0}{\int }}\overset{+\infty }{\underset{-\infty }{\int }}\left(\left(\rho u\right)\phi_{t}+\left(\rho u^{2}\right)\phi_{x}\right)\text{d}x\;\;\text{d}t=0, \end{array}$$

for any test function *ϕ* ∈ *C*_0_^*∞*^(*R* ^+^ × *R*).

Moreover, we define a two-dimensional weighted delta functions as follows.

**Definition 2**

A two-dimensional weighted delta function *w*(*s*)*δ*_*l*_ supported on a smooth curve *L* parameterized as *t* = *t*(*s*), *x* = *x*(*s*)  (*c* ≤ *s* ≤ *d*) is defined by

(15) $$\begin{array}{c} \langle w\left(s\right)\delta_{l},\phi \rangle =\overset{d}{\underset{c}{\int }}w\left(s\right)\phi \left(t\left(s\right),x\left(s\right)\right)\text{d}s, \end{array}$$

for all test functions *ϕ* ∈ *C*_0_^*∞*^(*R* ^+^ × *R*).

With these definitions, one can construct a *δ*-measure solution as

(16) $$\begin{array}{c} \left(u,\rho \right)\left(t,x\right)=\{\begin{array}{cc} \left(u_{-},\rho_{-}\right), & x<u_{\delta }t,\\ \left(u_{\delta },\omega \left(t\right)\delta \left(x-u_{\delta }t\right)\right), & x=u_{\delta }t,\\ \left(u_{+},\rho_{+}\right), & x>u_{\delta }t, \end{array} \end{array}$$

where *ω*(*t*) and *u*_*δ*_ are weight and velocity of the delta shock wave, respectively, satisfying the generalized Rankine-Hugoniot condition:

(17) dx(t)dt=uδ,dω(t)dt=uδ[ρ]−[ρu],dω(t)uδdt=uδ[ρu]−[ρu2],

with initial data *ω*(0) = 0, where [*ρ*] = *ρ*_+_ − *ρ*_−_. By simple calculation, we obtain

(18) uδ=ρ+u++ρ−u−ρ++ρ−,ω(t)=ρ−ρ+(u−−u+)t,

for *ρ*_−_ ≠ *ρ*_+_, and

(19) uδ=u+−u−2,ω(t)=ρ+(u−−u+)t,

for *ρ*_−_ = *ρ*_+_.

We can also justify that the delta shock wave satisfies the entropy condition:

(20) $$\begin{array}{c} u_{+}<u_{\delta }<u_{-}, \end{array}$$

which means that all the characteristics on both sides of the delta shock are incoming.

## 3. The Riemann Solutions for System (9)

In this section, we analyze some basic properties and solve the Riemann problem for (9).

### 3.1. The Riemann Solutions for System (9) and (10)

System (9) and (10) have two eigenvalues

(21) $$\begin{array}{c} \lambda_{1}=u-\epsilon \left(\gamma +1\right)\rho^{\gamma },\;\;\lambda_{2}=u-\epsilon \rho^{\gamma }, \end{array}$$

with corresponding right eigenvectors

(22) $$\begin{array}{c} r_{1}={\left(1,0\right)}^{T},\;\;r_{2}={\left(\rho ,\epsilon \gamma \rho^{\gamma }\right)}^{T}, \end{array}$$

satisfying

(23) $$\begin{array}{c} \nabla \lambda_{1}\cdot r_{1}=-\epsilon \gamma \left(\gamma +1\right)\rho^{\gamma -1}\neq 0,\;\;\nabla \lambda_{2}\cdot r_{2}=0. \end{array}$$

So the 1-characteristic field is genuinely nonlinear, and the 2-characteristic field is always linearly degenerate.

Since (9)-(10) and (12) remain invariant under a uniform expansion of coordinates *t* → *βt* and *x* → *βx*, *β* > 0, the solution is only connected with *ξ* = *x*/*t*. Thus we should seek the self-similar solution

(24) $$\begin{array}{c} \left(u,\rho \right)\left(x,t\right)=\left(u,\rho \right)\left(\xi \right),\;\xi =\frac{x}{t}. \end{array}$$

Then, the Riemann problem (9)-(10) and (12) can be reduced to

(25) $$\begin{array}{c} -\xi \rho_{\xi }+{\left(\rho \left(u-\epsilon \rho^{\gamma }\right)\right)}_{\xi }=0,\\ -\xi {\left(\rho u\right)}_{\xi }+{\left(\rho u\left(u-\epsilon \rho^{\gamma }\right)\right)}_{\xi }=0, \end{array}$$

with (*u*, *ρ*)(±*∞*) = (*u*_±_, *ρ*_±_).

For smooth solutions, system (25) can be rewritten as

(26) $$\begin{array}{c} \left(\begin{array}{cc} u-\epsilon \left(\gamma +1\right)\rho^{\gamma }-\xi & \rho \\ 0 & u-\epsilon \rho^{\gamma }-\xi \end{array}\right)\left(\begin{array}{c} \text{d}\rho \\ \text{d}u \end{array}\right)=0, \end{array}$$

which provides either the general solutions (constant states),

(27) $$\begin{array}{c} \left(u,\rho \right)\left(\xi \right)=\text{const},\;\left(\rho >0\right), \end{array}$$

or rarefaction wave, which is wave of the first characteristic family,

(28) $$\begin{array}{c} R:\{\begin{array}{cc} \xi =u-\epsilon \left(\gamma +1\right)\rho^{\gamma }, & \\ u=u_{-},\;\;\rho <\rho_{-}, & \end{array} \end{array}$$

or contact discontinuity, which is of the second characteristic family,

(29) $$\begin{array}{c} J\text{:}\{\begin{array}{cc} \xi =u-\epsilon \rho^{\gamma }, & \\ u=u_{-}+\epsilon \left(\rho^{\gamma }-\rho_{-}^{\gamma }\right). & \end{array} \end{array}$$

For a bounded discontinuity at *ξ* = *σ*_*ϵ*_, the Rankine-Hugoniot condition

(30) $$\begin{array}{c} -\sigma_{\epsilon }\left[\rho \right]\;+\left[\rho \left(u-\epsilon \rho^{\gamma }\right)\right]=0,\\ -\sigma_{\epsilon }\left[\rho u\right]+\left[\rho u\left(u-\epsilon \rho^{\gamma }\right)\right]=0, \end{array}$$

holds, where [*ρ*] = *ρ* − *ρ*_−_ and *σ*_*ϵ*_ is the velocity of the discontinuity. From (30), we obtain either shock wave, which is wave of the first characteristic family,

(31) $$\begin{array}{c} S\text{:}\{\begin{array}{cc} \sigma_{\epsilon }=u-\frac{\epsilon \left(\rho^{\gamma +1}-\rho_{-}^{\gamma +1}\right)}{\rho -\rho_{-}}, & \\ u=u_{-},\;\;\rho >\rho_{-}, & \end{array} \end{array}$$

or contact discontinuity, which is of the second characteristic family,

(32) $$\begin{array}{c} J\text{:}\{\begin{array}{cc} \sigma_{\epsilon }=u-\epsilon \rho^{\gamma }, & \\ u=u_{-}+\epsilon \left(\rho^{\gamma }-\rho_{-}^{\gamma }\right). & \end{array} \end{array}$$

Here we notice that the shock wave curve and the rarefaction wave curve passing through the same point (*u*_−_, *ρ*_−_) coincid in the phase plane; that is, (9)-(10) belong to “Temple class” [24].

**Figure 1:**
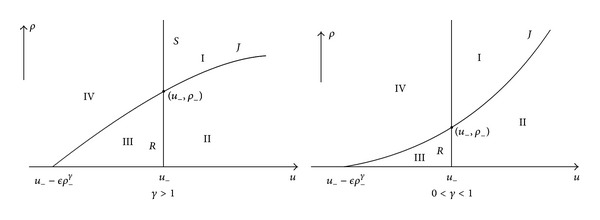
The upper half (*u*, *ρ*) plane with *p* = *ρ* ^*γ*^ is divided into 4 regions for both cases *γ* > 1 and 0 < *γ* < 1.

Through the point (*u*_−_, *ρ*_−_), we draw the curve *u* = *u*_−_ for *ρ* > 0 in the phase plane, which is parallel to the *ρ*-axis. We denote it by *R* when *ρ* < *ρ*_−_ and *S* when *ρ* > *ρ*_−_. Through the point (*u*_−_, *ρ*_−_), we draw the curve (29) which intersects the *u*-axis at the point (*u*_−_ − *ϵρ*_−_^*γ*^, 0), denoted by *J*. Then the phase plane is divided into four regions (see Figure 1). Thus we can construct the Riemann solutions of system (9)-(10) as follows:

1. when (*u*_+_, *ρ*_+_) ∈ I(*u*_−_, *ρ*_−_), that is, *u*_+_ > *u*_−_  and  *u*_+_ < *u*_−_ + *ϵ*(*ρ* ^*γ*^ − *ρ*_−_^*γ*^), the solution is *S* + *J*;
2. when (*u*_+_, *ρ*_+_) ∈ II(*u*_−_, *ρ*_−_), that is, *u*_+_ > *u*_−_  and  *u*_+_ > *u*_−_ + *ϵ*(*ρ* ^*γ*^ − *ρ*_−_^*γ*^), the solution is *R* + *J*;
3. when (*u*_+_, *ρ*_+_) ∈ III(*u*_−_, *ρ*_−_), that is, *u*_+_ < *u*_−_  and  *u*_+_ > *u*_−_ + *ϵ*(*ρ* ^*γ*^ − *ρ*_−_^*γ*^), the solution is *R* + *J*;
4. when (*u*_+_, *ρ*_+_) ∈ IV(*u*_−_, *ρ*_−_), that is, *u*_+_ < *u*_−_  and  *u*_+_ < *u*_−_ + *ϵ*(*ρ* ^*γ*^ − *ρ*_−_^*γ*^), the solution is *S* + *J*.

### 3.2. The Riemann Solutions of System (9) and (11)

Systems (9) and (11) have two eigenvalues:

(33) $$\begin{array}{c} \lambda_{1}=u+\epsilon \left(1-\alpha \right)\rho^{-\alpha },\;\;\lambda_{2}=u+\epsilon \rho^{-\alpha }, \end{array}$$

with corresponding right eigenvectors:

(34) $$\begin{array}{c} r_{1}={\left(1,0\right)}^{T},\;\;r_{2}={\left(\rho ,\epsilon \alpha \rho^{-\alpha }\right)}^{T}, \end{array}$$

satisfying

(35) $$\begin{array}{c} \nabla \lambda_{1}\cdot r_{1}=-\epsilon \alpha \left(1-\alpha \right)\rho^{-\alpha -1},\;\;\nabla \lambda_{2}\cdot r_{2}=0. \end{array}$$

Thus the 1-characteristic field is genuinely nonlinear and 2-characteristic field is always linearly degenerate as 0 < *α* < 1, while both the two characteristic fields are fully linearly degenerate as *α* = 1.

When 0 < *α* < 1, we get rarefaction wave and shock wave which can be expressed by

(36) $$\begin{array}{c} R\text{:}\{\begin{array}{cc} \xi =u+\epsilon \left(1-\alpha \right)\rho^{-\alpha }, & \\ u=u_{-},\;\;\rho <\rho_{-}, & \end{array}\\ S\text{:}\{\begin{array}{cc} \sigma_{\epsilon }=u+\frac{\epsilon \left(\rho^{1-\alpha }-\rho_{-}^{1-\alpha }\right)}{\rho -\rho_{-}}, & \\ u=u_{-},\;\;\rho >\rho_{-}, & \end{array} \end{array}$$

or contact discontinuity which can be expressed by

(37) $$\begin{array}{c} J\text{:}\{\begin{array}{cc} \tau_{\epsilon }=u+\epsilon \rho^{-\alpha }, & \\ u=u_{-}+\epsilon \left(\rho_{-}^{-\alpha }-\rho^{-\alpha }\right). & \end{array} \end{array}$$

**Figure 2:**
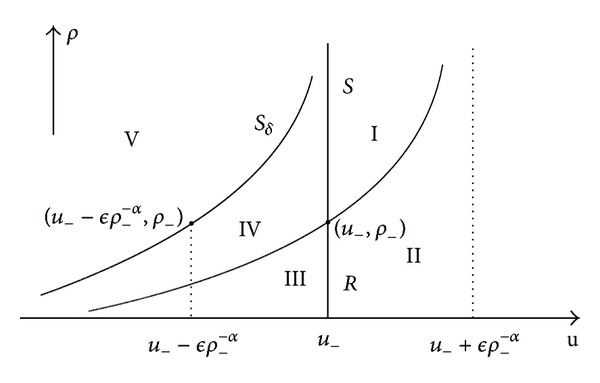
The upper half (*u*, *ρ*) plane with *p* = *ρ* ^*α*^  (0 < *α* < 1) is divided into 5 regions.

When 0 < *α* < 1, through the point (*u*_−_, *ρ*_−_), we draw the curve *u* = *u*_−_ for *ρ* > 0 in the phase plane, denoted by *R* when *ρ* < *ρ*_−_ and *S* when *ρ* > *ρ*_−_. Through the point (*u*_−_, *ρ*_−_), we draw the curve (37) which has two asymptotes *u* = *u*_−_ + *ϵρ*_−_^−*α*^ and *ρ* = 0, denoted by *J*. Through the point (*u*_−_ − *ϵ*/*ρ*_−_^*α*^, *ρ*_−_), we draw the curve (37), which has two asymptotic lines *u* = *u*_−_ and *ρ* = 0, denoted by *S*_*δ*_. Then the phase plane is divided into five regions; see Figure 2.

For any given (*u*_−_, *ρ*_−_), the Riemann solution is showed as follows:

1. when (*u*_+_, *ρ*_+_) ∈ I(*u*_−_, *ρ*_−_), that is, *u*_+_ > *u*_−_ and *u*_+_ < *u*_−_ + *ϵ*(*ρ*_−_^−*α*^ − *ρ* ^−*α*^), the solution is *S* + *J*;
2. when (*u*_+_, *ρ*_+_) ∈ II(*u*_−_, *ρ*_−_), that is, *u*_+_ > *u*_−_ and *u*_+_ > *u*_−_ + *ϵ*(*ρ*_−_^−*α*^ − *ρ* ^−*α*^), the solution is *R* + *J*;
3. when (*u*_+_, *ρ*_+_) ∈ III(*u*_−_, *ρ*_−_), that is, *u*_+_ < *u*_−_ and *u*_+_ > *u*_−_ + *ϵ*(*ρ*_−_^−*α*^ − *ρ* ^−*α*^), the solution is *R* + *J*;
4. when (*u*_+_, *ρ*_+_) ∈ IV(*u*_−_, *ρ*_−_), that is, *u*_+_ < *u*_−_ and *u*_+_ < *u*_−_ + *ϵ*(*ρ*_−_^−*α*^ − *ρ* ^−*α*^), the solution is *S* + *J*.

The nonvacuum intermediate constant state (*u*_∗_, *ρ*_∗_) is given by

(38) $$\begin{array}{c} \left(u_{*},\rho_{*}\right)=\left(u_{-},\sqrt[{\alpha }]{\frac{\epsilon }{u_{+}-u_{-}+\epsilon \rho_{+}^{-\alpha }}}\right). \end{array}$$

When (*u*_+_, *ρ*_+_) ∈ V(*u*_−_, *ρ*_−_), we introduce a definition of *δ*-measure solution, in which we introduce a definition of a generalized solution [19, 20, 22, 25] for system (9) and (11).

Suppose that Γ = {*γ*_*i*_ | *i* ∈ *I*} is a graph in the closed upper half-plane {(*x*, *t*) | *x* ∈ ℝ, *t* ∈ [0, +*∞*)} ⊂ ℝ^2^ containing smooth arcs *γ*_*i*_, *i* ∈ *I*, and *I* is a finite set. *I*_0_ is subset of *I* such that an arc *γ*_*k*_ for *k* ∈ *I*_0_ starts from the point of the *x*-axis; Γ_0_ = {*x*_*k*_^0^ | *k* ∈ *I*_0_} is the set of initial points of arc *γ*_*k*_, *k* ∈ *I*_0_.

Consider the *δ*-shock wave type initial data (*u* ^0^(*x*), *ρ* ^0^(*x*)), where

(39) $$\begin{array}{c} \rho^{0}\left(x\right)=\rho_{0}\left(x\right)+w^{0}\delta \left(\Gamma_{0}\right), \end{array}$$

*u* ^0^, *ρ*_0_ ∈ *L* ^*∞*^(ℝ; ℝ), *w* ^0^ *δ*(Γ_0_) = ∑_*k*∈*I*_0__ *w*_*k*_^0^ *δ*(*x* − *x*_*k*_^0^), and *w*_*k*_^0^ are constants for *k* ∈ *I*_0_. Furthermore, the pressure *p* = −*ρ* ^−*α*^ in (11) is a nonlinear term with respect to *ρ* defined by *p* ^0^(*x*, *t*) = −*ρ*_0_^−*α*^.

**Definition 3**

A pair of distributions (*u*(*x*, *t*), *ρ*(*x*, *t*)) and a graph Γ, where *ρ*(*x*, *t*) and *p*(*x*, *t*) have the form

(40) $$\begin{array}{c} \rho \left(x,t\right)=\bar{\rho }\left(x,t\right)+w\left(x,t\right)\delta \left(\Gamma \right),\;\;p\left(x,t\right)=-\rho {\left(x,t\right)}^{-\alpha }, \end{array}$$

$u,\bar{\rho }\in L^{\infty }(\mathbb{R}\times {\mathbb{R}}_{+};\mathbb{R}),w(x,t)\delta (\Gamma )=\sum_{i\in I}w_{i}(x,t)\delta (\gamma_{i}),w_{i}(x,t)\in C(\Gamma )$ for *i* ∈ *I* is called a generalized *δ*-shock wave type solution of system (9) with the initial data (*u* ^0^(*x*), *ρ* ^0^(*x*)) if the integral identities

(41) ∫0+∞∫−∞+∞(ρ¯ϕt+ρ¯(u−ϵp)ϕx)dx dt  +∑i∈I∫γiωi(x,t)∂ϕ∂ldl  +∫−∞+∞ρ0(x)ϕ(x,0)dx+∑k∈I0wk0ϕ(xk0,0)=0,∫0+∞∫−∞+∞(ρ¯uϕt+ρ¯u(u−ϵp)ϕx)dx dt  +∑i∈I∫γiwi(x,t)uδ(x,t)∂ϕ∂ldl  +∫−∞+∞ρ0(x)u0(x)ϕ(x,0)dx  +∑k∈I0wk0uδ0(xk0)ϕ(xk0,0)=0

hold for any test functions *ϕ*(*x*, *t*) ∈ *𝒟*(ℝ × ℝ_+_), where ∂*ϕ*/∂*l* is the tangential derivative on the graph Γ, ∫_*γ*_*i*__ *dl* is a line integral along the arc *γ*_*i*_, *u*_*δ*_(*x*, *t*) is the velocity of the *δ*-shock wave, and *u*_*δ*_^0^(*x*_*k*_^0^) = *u*_*δ*_(*x*_*k*_^0^, 0), *k* ∈ *I*_0_.

**Theorem 4**

When (*u*_+_, *ρ*_+_) ∈ *V*, for the Riemann problem (9), (11), and (12), there is a *δ*-shock wave solution (*u*(*x*, *t*), *ρ*(*x*, *t*)) with form

(42) u(x,t)=u−+[u]H(x−x(t)),ρ(x,t)=ρ−+[ρ]H(x−x(t))+w(t)δ(x−x(t)),

which satisfies the integral identities (41) in the sense of Definition 3, where Γ = {(*x*, *t*) | *x* = *x*(*t*) = *σt*, *t* ≥ 0}, $\bar{\rho }(x,t)=\rho_{-}+[\rho ]H(x-x(t))$,

(43) $$\begin{array}{c} \int_{\Gamma }w\left(x,t\right)\frac{\partial \phi \left(x,t\right)}{\partial l}=\overset{\infty }{\underset{0}{\int }}w\left(x,t\right)\frac{d\phi \left(x,t\right)}{dt}, \end{array}$$

and *H*(*x*) is the Heaviside function *H*(*x*) = 0(1), *x* < (>)0.

Suppose that *Ω* ⊂ ℝ × ℝ_+_ is a region cut by a smooth curve Γ = {(*x*, *t*) | *x* = *x*(*t*)} into left and right hand parts *Ω*_±_ = {(*x*, *t*) | ±(*x* − *x*(*t*)) > 0}; (*u*(*x*, *t*), *ρ*(*x*, *t*)) is a generalized *δ*-shock wave solution of system (9) and (11); functions $\bar{\rho }(x,t)$ and *u*(*x*, *t*) are smooth in *Ω*_±_ and have one-side limits ${\bar{\rho }}_{\pm },u_{\pm }$ on the curve Γ. Then the generalized Rankine-Hugoniot conditions for *δ*-shock wave are

(44) dx(t)dt=uδ,dω(t)dt=uδ[ρ]−[ρ(u+ϵρ−α)],d(ω(t)uδ)dt=uδ[ρu]−[ρu(u+ϵρ−α)],

with initial data *ω*(0) = 0, where [*ρ*] = *ρ*_+_ − *ρ*_−_, 0 < *α* < 1.

From (44), we obtain

(45) uδ=([2ρu+ϵρ1−α]+[2ρu+ϵρ1−α]2−4[ρ][ρu(u+ϵρ−α)])×(2[ρ])−1,ω(t)=((−[ϵρ1−α]+[2ρu+ϵρ1−α]2−4[ρ][ρu(u+ϵρ−α)])×(2)−1)t,

as *ρ*_−_ ≠ *ρ*_+_, and

(46) uδ=u+−u−+ϵρ+−α2,ω(t)=ρ+(u−−u+)t,

as *ρ*_−_ = *ρ*_+_.

We also can justify that the delta shock wave satisfies the entropy condition:

(47) $$\begin{array}{c} \lambda_{2}\left(u_{+},\rho_{+}\right)\leq u_{\delta }\leq \lambda_{1}\left(u_{-},\rho_{-}\right), \end{array}$$

which means that all the characteristics on both sides of the delta shock are not outcoming.

When *α* = 1, the detailed study can be found in [7]; we omit it.

Thus, we have obtained the solutions of the Riemann problem for (9).

## 4. Limit of Riemann Solutions to the Keyfitz-Kranzer Type System

In this section, our main purpose is to consider the limits of the Riemann solutions of (9) and compare them with the corresponding Riemann solutions to transport equations (8). Our discussion depends on the order of *u*_−_ and *u*_+_.

### 4.1. The Limits of Riemann Solutions of (9)-(10)

Firstly, we display the limit of Riemann solution to (9)-(10) for *u*_−_ < *u*_+_.

**Lemma 5**

In the case *u*_−_ < *u*_+_, when *ρ*_−_ ≥ *ρ*_+_, (*u*_+_, *ρ*_+_) ∈ II (*u*_−_, *ρ*_−_) for arbitrary *ϵ*; when *ρ*_−_ < *ρ*_+_, there exists *ϵ*_0_ = (*u*_+_ − *u*_−_)/(*ρ*_+_^*γ*^ − *ρ*_−_^*γ*^) > 0, such that (*u*_+_, *ρ*_+_) ∈ II (*u*_−_, *ρ*_−_) when 0 < *ϵ* < *ϵ*_0_.

This lemma shows that the curve *J* becomes steeper as *ϵ* is much small. As *u*_−_ < *u*_+_, from Lemma 5, we know that (*u*_+_, *ρ*_+_) ∈ II(*u*_−_, *ρ*_−_) when 0 < *ϵ* < *ϵ*_0_. Then the Riemann solutions of (9)-(10) consist of the rarefaction wave *R* and the contact discontinuity *J* with the intermediate constant state (*u*_∗_, *ρ*_∗_) besides the two constant states (*u*_±_, *ρ*_±_) as this form:

(48) $$\begin{array}{c} \left(u^{\epsilon },\rho^{\epsilon }\right)\left(\xi \right)=\{\begin{array}{cc} \left(u_{-},\rho_{-}\right), & -\infty <\xi \leq \lambda_{1}\left(u_{-},\rho_{-}\right),\\ R, & \lambda_{1}\left(u_{-},\rho_{-}\right)\leq \xi \leq \lambda_{1}\left(u_{*},\rho_{*}\right),\\ \left(u_{*},\rho_{*}\right), & \lambda_{1}\left(u_{*},\rho_{*}\right)\leq \xi <\tau_{\epsilon }\\ \left(u_{+},\rho_{+}\right), & \tau_{\epsilon }<\xi <+\infty , \end{array} \end{array}$$

where *λ*_1_ is determined by (21),

(49) $$\begin{array}{c} \tau_{\epsilon }=u_{+}-\epsilon \rho_{+}^{\gamma }, \end{array}$$

(50) $$\begin{array}{c} \left(u_{*},\rho_{*}\right)=\left(u_{-},\sqrt[{\gamma }]{\frac{u_{-}-u_{+}}{\epsilon }+\rho_{+}^{\gamma }}\right). \end{array}$$

When *u*_−_ < *u*_+_, from (50), and when *ϵ* is small enough to satisfy 0 < *ϵ* ≤ (*u*_+_ − *u*_−_)/*ρ*_+_^*γ*^, we know that a vacuum state appears in the Riemann solutions of (9)-(10). By (21), (49), and (50), it is easy to get that

(51) $$\begin{array}{c} \underset{\epsilon \to 0}{\lim}\lambda_{1}\left(u_{-},\rho_{-}\right)=\underset{\epsilon \to 0}{\lim}\left(u_{-}-\epsilon \left(\gamma +1\right)\rho_{-}^{\gamma }\right)=u_{-},\\ \underset{\epsilon \to 0}{\lim}\lambda_{1}\left(u_{*},\rho_{*}\right)=\underset{\epsilon \to 0}{\lim}\left(u_{*}-\epsilon \left(\gamma +1\right)\rho_{*}^{\gamma }\right)=u_{-},\\ \underset{\epsilon \to 0}{\lim}\tau_{\epsilon }=\underset{\epsilon \to 0}{\lim}\left(u_{+}-\epsilon \rho_{+}^{\gamma }\right)=u_{+}, \end{array}$$

which mean that the rarefaction wave *R* and the contact discontinuity *J*:  *u*_∗_ − *ϵρ*_∗_^*γ*^ = *u*_+_ − *ϵρ*_+_^*γ*^ become the contact discontinuities *J*_1_:  *u* = *u*_−_ and *J*_2_:  *u* = *u*_+_, respectively, as *ϵ* → 0. Meanwhile the vacuum state will fill up the region between the two contact discontinuities, which is exactly identical with the corresponding Riemann solutions of system (8).

Secondly, when *u*_+_ = *u*_−_, the Riemann solution contains a shock wave *S* with the propagating speed *σ*_*ϵ*_ besides the states (*u*_±_, *ρ*_±_) for *ρ*_+_ > *ρ*_−_, or a rarefaction wave *R* with the speed *λ*_1_(*u*, *ρ*)  (*ρ*_−_ ≥ *ρ* ≥ *ρ*_+_) for *ρ*_+_ < *ρ*_−_; see Figure 1. From (31) and (50), we obtain

(52) $$\begin{array}{c} \underset{\epsilon \to 0}{\lim}\sigma_{\epsilon }=\underset{\epsilon \to 0}{\lim}\left(u-\frac{\epsilon \left(\rho^{\gamma +1}-\rho_{-}^{\gamma +1}\right)}{\rho -\rho_{-}}\right)=u_{-}, \end{array}$$

or from (21) and (50), we have

(53) lim⁡ϵ→0λ1(u−,ρ−)=lim⁡ϵ→0(u−−ϵ(γ+1)ρ−γ)=lim⁡ϵ→0λ1(u+,ρ+)=lim⁡ϵ→0(u+−ϵ(γ+1)ρ+γ)=u−.

We conclude that, when *u*_−_ = *u*_+_, the Riemann solution of system (9)-(10) containing one shock wave or one rarefaction wave converges to the contact discontinuity solution of the transport equations (8) as *ϵ* → 0.

Finally, we display the limit of Riemann solutions to (9)-(10) for *u*_−_ > *u*_+_.

**Lemma 6**

In the case *u*_−_ > *u*_+_, when *ρ*_−_ ≤ *ρ*_+_, (*u*_+_, *ρ*_+_) ∈ IV (*u*_−_, *ρ*_−_) for arbitrary *ϵ*; when *ρ*_−_ > *ρ*_+_, there exists *ϵ*_1_ = (*u*_−_ − *u*_+_)/(*ρ*_−_^*γ*^ − *ρ*_+_^*γ*^) > 0, such that (*u*_+_, *ρ*_+_) ∈ IV (*u*_−_, *ρ*_−_) when 0 < *ϵ* < *ϵ*_1_.

From this lemma we know that the contact discontinuity *J* becomes steeper and steeper when *ϵ* decreases; that is, (*u*_+_, *ρ*_+_) ∈ IV(*u*_−_, *ρ*_−_) for small *ϵ*. In this case, the Riemann solution of (9)-(10) consists of a shock wave *S* and a contact discontinuity *J* with the intermediate constant state (*u*_∗_, *ρ*_∗_) as

(54) $$\begin{array}{c} \left(u^{\epsilon },\rho^{\epsilon }\right)=\{\begin{array}{cc} \left(u_{-},\rho_{-}\right), & -\infty <\xi <\sigma_{\epsilon },\\ \left(u_{*},\rho_{*}\right), & \sigma_{\epsilon }<\xi <\tau_{\epsilon },\\ \left(u_{+},\rho_{+}\right), & \tau_{\epsilon }<\xi <+\infty , \end{array} \end{array}$$

where (*u*_∗_, *ρ*_∗_) is given by (50) and

(55) $$\begin{array}{c} \sigma_{\epsilon }=u_{-}-\frac{\epsilon \left(\rho_{*}^{\gamma +1}-\rho_{-}^{\gamma +1}\right)}{\rho_{*}-\rho_{-}}. \end{array}$$

When *u*_−_ > *u*_+_, from (50), it is easy to see that

(56) $$\begin{array}{c} \underset{\epsilon \to 0}{\lim}\rho_{*}=\underset{\epsilon \to 0}{\lim}\sqrt[{\gamma }]{\frac{u_{-}-u_{+}}{\epsilon }+\rho_{+}^{\gamma }}=\infty . \end{array}$$

By (55), we obtain

(57) $$\begin{array}{c} \underset{\epsilon \to 0}{\lim}\sigma_{\epsilon }=\underset{\epsilon \to 0}{\lim}\left(u_{-}-\frac{\epsilon \left(\rho_{*}^{\gamma +1}-\rho_{-}^{\gamma +1}\right)}{\rho_{*}-\rho_{-}}\right)=u_{+}. \end{array}$$

From (56)-(57) and

(58) $$\begin{array}{c} \underset{\epsilon \to 0}{\lim}\tau_{\epsilon }=\underset{\epsilon \to 0}{\lim}\left(u_{+}-\epsilon \rho_{+}^{\gamma }\right)=u_{+}, \end{array}$$

we know that *S* and *J* coincide with a new type of nonlinear hyperbolic wave which is called the delta shock wave in [23]. Compared with the corresponding Riemann solutions of (8), it is clear to see that the propagation speed of the delta shock wave here is *u*_*δ*_ = *u*_+_ which is different from that of (8).

From (30), we have

(59) $$\begin{array}{c} \sigma_{\epsilon }\left(\rho_{*}-\rho_{-}\right)=\rho_{*}\left(u_{*}-\epsilon \rho_{*}^{\gamma }\right)-\rho_{-}\left(u_{-}-\epsilon \rho_{-}^{\gamma }\right),\\ \tau_{\epsilon }\left(\rho_{+}-\rho_{*}\right)=\rho_{+}\left(u_{+}-\epsilon \rho_{+}^{\gamma }\right)-\rho_{*}\left(u_{*}-\epsilon \rho_{*}^{\gamma }\right), \end{array}$$

which mean that

(60) $$\begin{array}{c} \underset{\epsilon \to 0}{\lim}\left(\sigma_{\epsilon }-\tau_{\epsilon }\right)\rho_{*}=u_{+}\left[\rho \right]-\left[\rho u\right]=\rho_{-}\left(u_{-}-u_{+}\right). \end{array}$$

It is obvious that

(61) $$\begin{array}{c} \omega \left(t\right)=\underset{\epsilon \to 0}{\lim}\overset{\tau_{\epsilon }t}{\underset{\sigma_{\epsilon }t}{\int }}\rho_{*}dx=\underset{\epsilon \to 0}{\lim}\left(\sigma_{\epsilon }-\tau_{\epsilon }\right)\rho_{*}t=\rho_{-}\left(u_{-}-u_{+}\right)t. \end{array}$$

From (61), we obtain that the strength of the delta shock wave is also different from transport equations (8), which may be due to the different propagation speed of the delta shock wave. For the limit situation of (9)-(10), the characteristics on the left side of the delta shock wave will come into the delta shock wave line *x* = *u*_+_ *t* while the characteristics on the right side of it will be parallel to it. For transport equations (8), the characteristics on the two sides will come into the delta shock wave curve *x* = *u*_*δ*_ *t*. So, the Riemann solution of (9)-(10) does not converge to solution of (8) as *ϵ* → 0 when *u*_−_ > *u*_+_.

### 4.2. The Limit of Riemann Solutions of System (9) and (11)

In this subsection, we deal with the limit behavior of Riemann solutions to system (9) and (11).

Firstly, we display the limit of Riemann solutions to (9) and (11) for *u*_−_ < *u*_+_.

**Lemma 7**

For the case *u*_−_ < *u*_+_, when *ρ*_−_ ≥ *ρ*_+_, (*u*_+_, *ρ*_+_) ∈ II (*u*_−_, *ρ*_−_) for arbitrary *ϵ*; when *ρ*_−_ < *ρ*_+_, then there exists *ϵ*_0_ = (*u*_+_ − *u*_−_)/(*ρ*_−_^−*α*^ − *ρ*_+_^−*α*^) > 0 such that (*u*_+_, *ρ*_+_) ∈ II (*u*_−_, *ρ*_−_) as 0 < *ϵ* < *ϵ*_0_.

From Lemma 7, we know that the contact discontinuity *J* becomes steeper as *ϵ* becomes smaller and smaller; that is, (*u*_+_, *ρ*_+_) ∈ II (*u*_−_, *ρ*_−_) for small *ϵ*. Then the Riemann solution of (9) and (11) consists of a rarefaction wave *R* and a contact discontinuity *J* with the intermediate constant state (*u*_∗_, *ρ*_∗_) besides the two constant states (*u*_±_, *ρ*_±_), which has this form:

(62) $$\begin{array}{c} \left(u^{\epsilon },\rho^{\epsilon }\right)=\{\begin{array}{cc} \left(u_{-},\rho_{-}\right), & -\infty <\xi <\lambda_{1}\left(u_{-},\rho_{-}\right),\\ R, & \lambda_{1}\left(u_{-},\rho_{-}\right)\leq \xi \leq \lambda_{1}\left(u_{*},\rho_{*}\right),\\ \left(u_{*},\rho_{*}\right), & \lambda_{1}\left(u_{*},\rho_{*}\right)<\xi <\tau_{\epsilon },\\ \left(u_{+},\rho_{+}\right), & \tau_{\epsilon }<\xi <+\infty , \end{array} \end{array}$$

where *λ*_1_, (*u*_∗_, *ρ*_∗_) are determined by (33) and (38), respectively, and

(63) $$\begin{array}{c} \tau_{\epsilon }=u_{+}+\epsilon \rho_{+}^{-\alpha }. \end{array}$$

From (38), we obtain

(64) $$\begin{array}{c} \underset{\epsilon \to 0}{\lim}\rho_{*}=\underset{\epsilon \to 0}{\lim}\sqrt[{\alpha }]{\frac{\epsilon }{u_{+}-u_{-}+\epsilon \rho_{+}^{-\alpha }}}=0, \end{array}$$

and then a vacuum state appears in the Riemann solution of (9)–(11).

By (33), (38), and (63), we get

(65) $$\begin{array}{c} \underset{\epsilon \to 0}{\lim}\lambda_{1}\left(u_{-},\rho_{-}\right)=\underset{\epsilon \to 0}{\lim}\lambda_{1}\left(u_{*},\rho_{*}\right)=u_{-},\\ \underset{\epsilon \to 0}{\lim}\tau_{\epsilon }=u_{+}, \end{array}$$

which mean that the rarefaction wave *R* and the contact discontinuity *J* become the contact discontinuities *J*_1_:  *u* = *u*_−_ and *J*_2_:  *u* = *u*_+_, respectively, as *ϵ* → 0. Meanwhile the vacuum state will fill up the region between the two contact discontinuities, which is exactly identical with the corresponding Riemann solution of system (8).

Secondly, when *u*_+_ = *u*_−_, as done in Section 4.1, it is easy to see that the Riemann solution of (9) and (11) converges to the contact discontinuity of system (8); we omit it.

Finally, we discuss the limit of Riemann solutions of (9) and (11) when *u*_−_ > *u*_+_.

**Lemma 8**

If *u*_−_ > *u*_+_, then there exist *ϵ*_1_, *ϵ*_2_ > 0 such that (*u*_+_, *ρ*_+_) ∈ IV (*u*_−_, *ρ*_−_) when *ϵ*_2_ < *ϵ* < *ϵ*_1_; (*u*_+_, *ρ*_+_) ∈ V (*u*_−_, *ρ*_−_) when 0 < *ϵ* < *ϵ*_2_.

**Proof**

When *ρ*_−_ ≤ *ρ*_+_, it is easy to find that (*u*_+_, *ρ*_+_) ∈ IV ∪ V(*u*_−_, *ρ*_−_) for arbitrary *ϵ* directly from Figure 2. On the other hand, when *ρ*_−_ > *ρ*_+_ and (*u*_+_, *ρ*_+_) ∈ IV ∪ V(*u*_−_, *ρ*_−_), see Figure 2 together with (37), we can get that *ϵ* should satisfy *u*_+_ + *ϵρ*_+_^−*α*^ < *u*_−_ + *ϵρ*_−_^−*α*^, which gives *ϵ* < (*u*_−_ − *u*_+_)/(*ρ*_+_^−*α*^ − *ρ*_−_^−*α*^). In one word, (*u*_+_, *ρ*_+_) ∈ IV ∪ V(*u*_−_, *ρ*_−_) for small *ϵ*.

If (*u*_+_, *ρ*_+_) ∈ IV(*u*_−_, *ρ*_−_), (*u*_+_, *ρ*_+_) should satisfy *u*_+_ < *u*_−_, *u*_+_ + *ϵρ*_+_^−*α*^ < *u*_−_ + *ϵρ*_−_^−*α*^, and *u*_+_ > *u*_−_ − *ϵρ*_+_^−*α*^. From the above inequalities, we obtain (*u*_+_, *ρ*_+_) ∈ IV(*u*_−_, *ρ*_−_) when *ϵ*_2_ < *ϵ* < *ϵ*_1_, and (*u*_+_, *ρ*_+_) ∈ V(*u*_−_, *ρ*_−_) when 0 < *ϵ* < *ϵ*_2_, where

(66) $$\begin{array}{c} \epsilon_{1}=\frac{u_{-}-u_{+}}{\rho_{+}^{-\alpha }-\rho_{-}^{-\alpha }},\;\;\epsilon_{2}=\left(u_{-}-u_{+}\right)\rho_{+}^{\alpha }. \end{array}$$

The results have been obtained.

When *u*_−_ > *u*_+_ and *ϵ*_2_ < *ϵ* < *ϵ*_1_, the Riemann solution of (9) and (11) consists of a shock wave *S* and a contact discontinuity *J* with the intermediate state (*u*_∗_, *ρ*_∗_) besides the two constant states (*u*_±_, *ρ*_±_), which is as this form:

(67) $$\begin{array}{c} \left(u^{\epsilon },\rho^{\epsilon }\right)=\{\begin{array}{cc} \left(u_{-},\rho_{-}\right), & -\infty <\xi <\sigma_{\epsilon },\\ \left(u_{*},\rho_{*}\right), & \sigma_{\epsilon }<\xi <\tau_{\epsilon },\\ \left(u_{+},\rho_{+}\right), & \tau_{\epsilon }<\xi <+\infty , \end{array} \end{array}$$

where (*u*_∗_, *ρ*_∗_), *τ*_*ϵ*_ are determined by (38) and (63), respectively, and

(68) $$\begin{array}{c} \sigma_{\epsilon }=u_{-}+\frac{\epsilon \left(\rho_{*}^{1-\alpha }-\rho_{-}^{1-\alpha }\right)}{\rho_{*}-\rho_{-}}. \end{array}$$

It is easy to see that

(69) $$\begin{array}{c} \epsilon \rho_{*}^{-\alpha }=u_{+}-u_{-}+\epsilon \rho_{+}^{-\alpha }. \end{array}$$

For given *ρ*_+_ > 0, letting *ϵ* → *ϵ*_2_ = (*u*_−_ − *u*_+_)*ρ*_+_^*α*^ in (69) yields

(70) $$\begin{array}{c} \underset{\epsilon \to \epsilon_{2}}{\lim}\epsilon \rho_{*}^{-\alpha }=\underset{\epsilon \to \epsilon_{2}}{\lim}\left(u_{+}-u_{-}+\epsilon \rho_{+}^{-\alpha }\right)=0. \end{array}$$

Hence, we deduce that

(71) $$\begin{array}{c} \underset{\epsilon \to \epsilon_{2}}{\lim}\rho_{*}=\infty . \end{array}$$

Thus we have the following result.

**Lemma 9**

Consider

(72) $$\begin{array}{c} \underset{\epsilon \to \epsilon_{2}}{\lim}u_{*}=\underset{\epsilon \to \epsilon_{2}}{\lim}\sigma_{\epsilon }=\underset{\epsilon \to \epsilon_{2}}{\lim}\tau_{\epsilon }=u_{-}, \end{array}$$

where *σ*_*ϵ*_, *τ*_*ϵ*_ is given by (63) and (68), and

(73) $$\begin{array}{c} \underset{\epsilon \to \epsilon_{2}}{\lim}\overset{\sigma_{\epsilon }t}{\underset{\tau_{\epsilon }t}{\int }}\rho_{*}dx=\left(u_{-}\left[\rho \right]-\left[\rho \left(u-\epsilon p\right)\right]\right)t. \end{array}$$

**Proof**

Due to (63) and (68), we get

(74) lim⁡ϵ→ϵ2σϵ=lim⁡ϵ→ϵ2(u−+ϵ(ρ∗1−α−ρ−1−α)ρ∗−ρ−)=u−,lim⁡ϵ→ϵ2τϵ=lim⁡ϵ→ϵ2(u++ϵρ+−α)=u−.

Thus it can be seen from (74) that shock wave *S* and contact discontinuity *J* will coalesce together when *ϵ* arrives at *ϵ*_2_.

Using the Rankine-Hugoniot condition for shock *S* and contact discontinuity *J*, we have

(75) $$\begin{array}{c} \sigma_{\epsilon }\left(\rho_{*}-\rho_{-}\right)=\rho_{*}\left(u_{*}+\epsilon \rho_{*}^{-\alpha }\right)-\rho_{-}\left(u_{-}+\epsilon \rho_{-}^{-\alpha }\right),\\ \tau_{\epsilon }\left(\rho_{+}-\rho_{*}\right)=\rho_{+}\left(u_{+}+\epsilon \rho_{+}^{-\alpha }\right)-\rho_{*}\left(u_{*}+\epsilon \rho_{*}^{-\alpha }\right), \end{array}$$

which implies that

(76) $$\begin{array}{c} \underset{\epsilon \to \epsilon_{2}}{\lim}\left(\sigma_{\epsilon }-\tau_{\epsilon }\right)\rho_{*}=\left(u_{-}\left[\rho \right]-\left[\rho \left(u-\epsilon p\right)\right]\right). \end{array}$$

It is obvious that

(77) $$\begin{array}{c} \underset{\epsilon \to \epsilon_{2}}{\lim}\overset{\tau_{\epsilon }t}{\underset{\sigma_{\epsilon }t}{\int }}\rho_{*}dx=\underset{\epsilon \to \epsilon_{2}}{\lim}\left(\sigma_{\epsilon }-\tau_{\epsilon }\right)\rho_{*}t=\left(u_{-}\left[\rho \right]-\left[\rho \left(u-\epsilon p\right)\right]\right)t. \end{array}$$

The proof is completed.

From Lemma 5, it can be concluded that the shock wave *S* and contact discontinuity *J* will coincide when *ϵ* tends to *ϵ*_2_. On the other hand, for *ρ*_+_ ≠ *ρ*_−_, by substituting *ϵ* = *ϵ*_2_ = (*u*_−_ − *u*_+_)*ρ*_+_^*α*^ into (45), we have

(78) uδ=u−,ω(t)=(uδ[ρ]−[ρ(u−ϵp)])t.

So, we obtain that the quantities *u*_*δ*_, *ω*(*t*) and the limits of *u*_∗_, *σ*_*ϵ*_ and *τ*_*ϵ*_ are consistent with (45) as proposed for the Riemann solutions of (9) and (11) for *ρ*_+_ ≠ *ρ*_−_ when we take *ϵ* = *ϵ*_2_. Otherwise, the assert is obviously true when *ρ*_+_ = *ρ*_−_. Thus, it uniquely determines that the limit of the Riemann solutions to system (9) and (11) when *ϵ* → *ϵ*_2_ in the case (*u*_+_, *ρ*_+_) ∈ IV(*u*_−_, *ρ*_−_) is just the delta shock solution of (9) and (11) in the case (*u*_+_, *ρ*_+_) ∈ *S*_*δ*_, where the curve *S*_*δ*_ is actually the boundary between the regions IV(*u*_−_, *ρ*_−_) and V(*u*_−_, *ρ*_−_).

**Theorem**

In the case *u*_−_ > *u*_+_, for each fixed *ϵ* ∈ (*ϵ*_2_, *ϵ*_1_), assume that (*u* ^*ϵ*^, *ρ* ^*ϵ*^) is a solution containing the shock wave *S* and contact discontinuity *J* of (9) and (11) with Riemann initial data, constructed in Section 3.2. Then, (*u* ^*ϵ*^, *ρ* ^*ϵ*^) converges in the sense of distributions, when *ϵ* → *ϵ*_2_, and the limit functions *ρ* and *ρu* are the sum of step function and a *δ*-measure with weights

(79) $$\begin{array}{c} \left(u_{\delta }\left[\rho \right]-\left[\rho \left(u-\epsilon p\right)\right]\right)t,\;\;\left(u_{\delta }\left[\rho u\right]-\left[\rho u\left(u-\epsilon p\right)\right]\right)t, \end{array}$$

respectively, and then form a delta shock solutions of (9) and (11) when *ϵ* → *ϵ*_2_.

**Proof**

When (*u*_+_, *ρ*_+_) ∈ IV(*u*_−_, *ρ*_−_), let *ξ* = *x*/*t*; then for each fixed *ϵ* > 0, the Riemann solutions are determined by

(80) $$\begin{array}{c} \left(u^{\epsilon },\rho^{\epsilon }\right)\left(\xi \right)=\{\begin{array}{cc} \left(u_{-},\rho_{-}\right), & -\infty <\xi <\sigma_{\epsilon },\\ \left(u_{*}^{\epsilon },\rho_{*}^{\epsilon }\right), & \sigma_{\epsilon }<\xi <\tau_{\epsilon },\\ \left(u_{+},\rho_{+}\right), & \tau_{\epsilon }<\xi <\infty , \end{array} \end{array}$$

which satisfy

(81) ∫−∞∞(ξ−(uϵ(ξ)−ϵp(ρϵ)))ρϵ(ξ)ϕ′(ξ)dξ +∫−∞∞ρϵ(ξ)ϕ(ξ)dξ=0,∫−∞∞(ξ−(uϵ(ξ)−ϵp(ρϵ)))ρϵ(ξ)uϵ(ξ)ϕ′(ξ)dξ +∫−∞∞ρϵ(ξ)uϵ(ξ)ϕ(ξ)dξ=0,

for any test function *ϕ* ∈ *C*_0_^*∞*^(−*∞*, *∞*).

The first integral in (81) can be decomposed into

(82) $$\begin{array}{c} \left\{\overset{\sigma_{\epsilon }}{\underset{-\infty }{\int }}+\overset{\tau_{\epsilon }}{\underset{\sigma_{\epsilon }}{\int }}+\overset{\infty }{\underset{\tau_{\epsilon }}{\int }}\right\}\left(\xi -\left(u^{\epsilon }\left(\xi \right)-\epsilon p\left(\rho^{\epsilon }\right)\right)\right)\rho^{\epsilon }\left(\xi \right)\phi^{\prime }\left(\xi \right)d\xi . \end{array}$$

The sum of the first and the last terms in (82) is

(83) ∫−∞σϵ(ξ−(u−−ϵp−))ρ−ϕ′(ξ)dξ +∫τϵ∞(ξ−(u+−ϵp+))ρ+ϕ′(ξ)dξ=−ρ−(u−−ϵp−)ϕ(σϵ)+ρ+(u+−ϵp+)ϕ(τϵ) +ρ−σϵϕ(σϵ)−ρ+τϵϕ(τϵ) −ρ−∫−∞σϵϕ(ξ)dξ−ρ+∫τϵ∞ϕ(ξ)dξ.

Letting *ϵ* → *ϵ*_2_ in (83), we have

(84) lim⁡ϵ→ϵ2(∫−∞σϵ+∫τϵ∞)(ξ−(uϵ(ξ)−ϵp(ρϵ)))ρϵ(ξ)ϕ′(ξ)dξ =([ρ(u−ϵp)]−uδ[ρ])ϕ(uδ)  −∫−∞∞ρ0(ξ−uδ)ϕ(ξ)dξ,

where *ρ*_0_(*ξ*) = *ρ*_−_ + [*ρ*]*H*(*ξ* − *σ*) and *H* is the Heaviside function.

The second term in (82) can be calculated by

(85) ∫σϵτϵ(ξ−(uϵ(ξ)−ϵp(ρϵ)))ρϵ(ξ)ϕ′(ξ)dξ =−ρ∗ϵ(u∗ϵ−ϵp(ρ∗ϵ))(ϕ(σϵ)−ϕ(τϵ))  −ρ∗ϵ∫σϵτϵϕξ dξ+ρ∗ϵ(τϵϕτϵ−σϵϕ(σϵ)).

By lim⁡_*ϵ*→*ϵ*_2__ *u*_∗_^*ϵ*^ = lim⁡_*ϵ*→*ϵ*_2__ *σ*_*ϵ*_ = lim⁡_*ϵ*→*ϵ*_2__ *τ*_*ϵ*_ = *u*_*δ*_ = *u*_−_, we obtain

(86) $$\begin{array}{c} \underset{\epsilon \to \epsilon_{2}}{\lim}\overset{\tau_{\epsilon }}{\underset{\sigma_{\epsilon }}{\int }}\left(\xi -\left(u^{\epsilon }\left(\xi \right)-\epsilon p\left(\rho^{\epsilon }\right)\right)\right)\rho^{\epsilon }\left(\xi \right)\phi^{\prime }\left(\xi \right)d\xi =0. \end{array}$$

Then, from (81)_1_, (84), and (86), we get that

(87) lim⁡ϵ→ϵ2∫−∞∞(ρϵ(ξ)−ρ0(ξ−uδ))ϕ(ξ)dξ  =(uδ[ρ]−[ρ(u−ϵp)])ϕ(uδ)

holds for any test function *ϕ* ∈ *C*_0_^*∞*^(−*∞*, *∞*).

With the same reason as above, we have

(88) lim⁡ϵ→ϵ2∫−∞∞(ρϵ(ξ)uϵ(ξ)−ρ0u0(ξ−uδ))ϕ(ξ)dξ  =(uδ[ρu]−[ρu(u−ϵp)])ϕ(uδ).

Finally, we study the limits of *ρ* ^*ϵ*^ and *ρ* ^*ϵ*^ *u* ^*ϵ*^ as *ϵ* → *ϵ*_2_, by tracing the time-dependence of weights of the *δ*-measure.

Let *φ*(*x*, *t*) ∈ *C*_0_^*∞*^((−*∞*, *∞*)×[0, *∞*)) and set $\tilde{\varphi }(\xi ,t):=\varphi (\xi t,t))$; then we obtain

(89) lim⁡ϵ→ϵ2∫0∞∫−∞∞ρϵ(xt)φ(x,t)dx dt  =lim⁡ϵ→ϵ2∫0∞t(∫−∞∞ρϵ(ξ)φ~(ξ,t)dξ)dt.

On the other hand,

(90) lim⁡ϵ→ϵ2∫−∞∞ρϵ(ξ)φ~(ξ,t)dξ =∫−∞∞ρ0(ξ−uδ)φ~(ξ,t)dξ  +(uδ[ρ]−[ρ(u−ϵp)])φ~(ξ,t) =t−1∫−∞∞ρ0(x−uδt)φ(x,t)dx  +(uδ[ρ]−[ρ(u−ϵp)])φ(uδt,t).

By (89) and (90), we get

(91) lim⁡ϵ→ϵ2∫0∞∫−∞∞ρϵ(xt)φ(x,t)dx dt=∫0∞∫−∞∞ρ0(x−uδt)φ(x,t)dx dt +∫0∞t(uδ[ρ]−[ρ(u−ϵp)])φ(x,t)dt.

With the same reason as before, we obtain

(92) lim⁡ϵ→ϵ2∫0∞∫−∞∞ρϵ(xt)uϵ(xt)φ(x,t)dx dt =∫0∞∫−∞∞ρ0u0(x−uδt)φ(x,t)dx dt  +∫0∞t(uδ[ρu]−[ρu(u−ϵp)])φ(x,t)dt.

Thus the result has been obtained.

When *u*_−_ > *u*_+_ and 0 < *ϵ* < *ϵ*_2_, (*u*_+_, *ρ*_+_) ∈ V(*u*_−_, *ρ*_−_). So the Riemann solution of (9) and (11) consists of a delta shock wave besides the constant states (*u*_±_, *ρ*_±_). We want to observe the behavior of strength and propagation speed of the delta shock wave when *ϵ* decreases and finally tends to zero.

For *ρ*_+_ ≠ *ρ*_−_, letting *ϵ* → 0 in (45), we have

(93) $$\begin{array}{c} \underset{\epsilon \to 0}{\lim}u_{\delta }\left(\epsilon \right)=\frac{\sqrt{\rho_{+}}u_{+}+\sqrt{\rho_{-}}u_{-}}{\sqrt{\rho_{+}}+\sqrt{\rho_{-}}},\\ \underset{\epsilon \to 0}{\lim}\omega \left(t,\epsilon \right)=\sqrt{\rho_{-}\rho_{+}}\left(u_{-}-u_{+}\right)t. \end{array}$$

For the special situation *ρ*_+_ = *ρ*_−_, by (46), we can obtain the same result as above.

From the above discussion, we can conclude that the limit of the strength and propagation speed of the delta shock wave in Riemann solution of system (9) and (11) are in accordance with those of transport equations (8) with the same Riemann initial data. That is to say, the delta shock solution to system (9) and (11) converges to the delta shock solution to transport equations (8) as pressure vanishes.

Combining the results of the above, when (*u*_+_, *ρ*_+_) ∈ IV(*u*_−_, *ρ*_−_), we conclude that the shock wave and a contact discontinuity coincide as a delta shock wave when *ϵ* → *ϵ*_2_. As *ϵ* continues to drop and goes to zero eventually, the delta shock solution is nothing but the Riemann solution to transport equations (8).

## 5. Conclusion

So far, the discussion for limit of Riemann solutions to the nonsymmetric system of Keyfitz-Kranzer type with both the polytropic gas and generalized Chaplygin gas has been completed. From the above analysis, as the pressure vanishes, there appear delta shock wave, vacuum state, and contact discontinuity when *u*_−_ > *u*_+_, *u*_−_ < *u*_+_, and *u*_−_ = *u*_+_, respectively. For the polytropic gas, different from cases of some other systems such as Euler equations or relativistic Euler equations, the delta shock wave is not the one of transport equations as parameter *ϵ* tends to zero. For the generalized Chaplygin gas, the delta shock wave appears as parameter *ϵ* tends to *ϵ*_2_, depending only on the Riemann initial data. Then as *ϵ* becomes smaller and goes to zero at last, the delta shock wave solution is the exact one of transport equations.
